# Triglyceride glucose-body mass index and the risk of diabetes: a general population-based cohort study

**DOI:** 10.1186/s12944-021-01532-7

**Published:** 2021-09-06

**Authors:** Xiaoyu Wang, Jingdong Liu, Zongyou Cheng, Yanjia Zhong, Xiaohua Chen, Wei Song

**Affiliations:** 1 Department of Endocrinology, Jiangxi Provincial People’s Hospital Affiliated to Nanchang University, 330006 Nanchang, Jiangxi Province China; 2 Department of Endocrinology, Yihuang County People’s Hospital, 344400 Fuzhou, Jiangxi Province China

**Keywords:** Diabetes, Cohort study, TyG-BMI, Risk factors, Triglyceride glucose-body mass index

## Abstract

**Background:**

Triglyceride glucose-body mass index (TyG-BMI) has been proven to be a reliable substitute for insulin resistance. However, whether a causal association exists between TyG-BMI and new-onset diabetes remains uncertain. The purpose of this study was to investigate the causal association and predictive performance between TyG-BMI and diabetes.

**Methods:**

A total of 116,661 subjects who underwent a physical examination were included in this study. The subjects were divided into five equal points according to the quintile of TyG-BMI, and the outcome of interest was the occurrence of diabetic events. TyG-BMI = ln [fasting plasma glucose (mg/dL) × fasting triglycerides (mg/dL)/2] × BMI.

**Results:**

During the average follow-up period of 3.1 (0.95) years, 1888 men (1.61 %) and 793 women (0.68 %) were newly diagnosed with diabetes. Multivariate Cox regression analysis showed that TyG-BMI was an independent predictor of new-onset diabetes (HR 1.50 per SD increase, 95 %CI: 1.40 to 1.60, *P*-trend < 0.00001), and the best TyG-BMI cutoff value for predicting new-onset diabetes was 213.2966 (area under the curve 0.7741, sensitivity 72.51 %, specificity 69.54 %). Additionally, the results of subgroup analysis suggested that the risk of TyG-BMI-related diabetes in young and middle-aged people was significantly higher than that in middle-aged and elderly people, and the risk of TyG-BMI-related diabetes in non-obese people was significantly higher than that in overweight and obese people (*P* for interaction < 0.05).

**Conclusions:**

This cohort study of the Chinese population shows that after excluding other confounding factors, there is a causal association of TyG-BMI with diabetes, and this independent association is more obvious in young, middle-aged and non-obese people.

**Supplementary Information:**

The online version contains supplementary material available at 10.1186/s12944-021-01532-7.

## Background

Diabetes is a metabolic disease caused by a disorder of blood glucose metabolism, and it is one of the most common chronic diseases in the world [1, 2]. In recent years, with the further aggravation of the global aging trend, the prevalence of obesity, and the drastic changes in traditional dietary patterns and lifestyles, the proportion of people with diabetes has increased rapidly [3, 4]. According to a recent survey by the International Diabetes Federation, 463 million people worldwide suffered from diabetes in 2019, including 116 million (30.45 %) in China, 101 million (21.81 %) in India and 34 million (21.3 %) in the United States. It is considered that the number of diabetics worldwide will reach 700 million by 2045 [5]. Diabetes can lead to microvascular and macrovascular complications, bring profound psychological and physical pain to patients, and impose a heavy burden on the health care system [5, 6]. Therefore, in view of the large number of patients with diabetes and the huge disease burden, it will be very meaningful to identify high-risk groups prone to diabetes through simple and effective diagnostic tools at an early stage.

Genetics, diet, lifestyle and environmental factors may all contribute to diabetes [3, 7]. Insulin resistance (IR) is the key mechanism of many metabolic diseases, such as diabetes, metabolic syndrome, obesity, and nonalcoholic fatty liver disease [1, 8–10]. It can better reflect the metabolic state of the body [11, 12]. However, at present, the main methods for detecting IR depend on the hyperinsulinemic-euglycemic clamp (HIEC) technique [13]. For the physical examination of the general population, if HIEC technology is used to detect IR, it will obviously increase the economic and time costs [14]. The triglyceride glucose (TyG) index is the product of fasting triglycerides (TG) and fasting plasma glucose (FPG), and a large number of studies in recent years have shown that the TyG index is a reliable substitute for IR [15–17]. There is recent evidence that a combination of the TyG index and body mass index (BMI) has better diagnostic value in differentiating IR [18]. Two cross-sectional studies further found a correlation between diabetes and TyG-BMI [19, 20]. However, it is not clear whether time progression affects the relationship between diabetes and TyG-BMI. Therefore, the aim of the present study was to examine the relationship between TyG-BMI and T2DM risk in a Chinese population and its predictive value to provide new ideas for the early detection and prevention of diabetes.

## Methods

### Study design and data source

This study was a post hoc analysis of a cohort study conducted by the China Rich Health Care Group, and the design has been detailed elsewhere [21]. In brief, the Rich Health Care Group Cohort Study recruited 685,277 adult subjects from 11 cities in China who participated in health examinations between 2011 and 2016. This project aims to promote the health of the Chinese population and assess diabetes and its risk factors through health examinations and follow-up of the general population. The available data for the study have been uploaded to the Dryad database by Professor Chen [22]. According to the Dryad database terms of service, researchers can freely use public data for postanalysis to make the data play a greater role. Due to the anonymity of the data and the research ethics having been approved in previous studies, there was no need to reapply for this study.

**Fig. 1:**
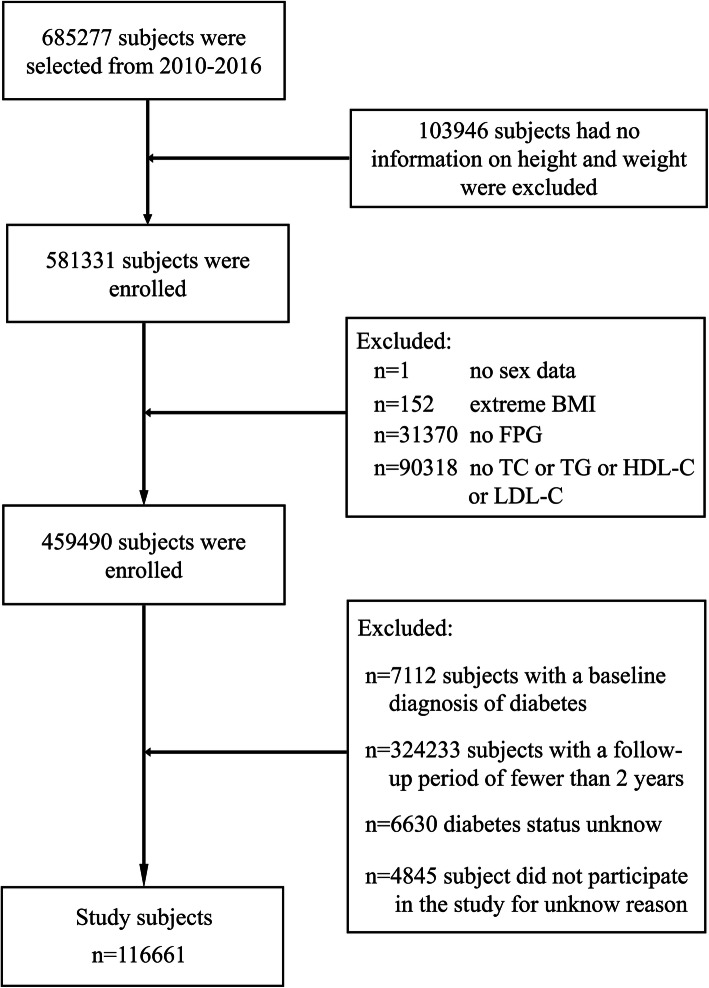
Flow diagram of subjects included in the cohort study

This study was a post hoc analysis based on previous research. According to the purpose of the study, the exposure factor of this study was set as TyG-BMI, and the outcome of interest was a new diabetes event. Research hypothesis: Can TyG-BMI be used to independently predict new diabetes events in the Chinese population? The exclusion criteria for the study subjects were as follows (Fig. 1): (1) subjects who had been diagnosed with diabetes at the time of the baseline interview; (2) unknown diabetes status during follow-up; (3) subjects with a follow-up period of less than 2 years; and (4) incomplete data or extreme values of sex, BMI, FPG and lipid parameters. (5) subjects without height or weight measurement information; and (6) subjects who did not participate in this study for unknown reasons. In this study, 116,661 subjects who met the criteria were finally analyzed.

### Data collection

All subjects were asked to complete a standardized questionnaire containing data on demographic characteristics and physical examination results, including age, family history of diabetes, sex, blood pressure, height, weight, and smoking/drinking status. Among them, weight and height were measured indoors, and the subjects wore no shoes but only light clothing. Blood pressure was measured in a quiet environment using a standard mercury sphygmomanometer. Fasting venous blood was collected by trained personnel and analyzed in a standard laboratory using an automated analyzer (Beckman 5800), in which the level of plasma glucose was measured by glucose oxidase method.

### Definition and calculation

The start time of follow-up was considered to be after the subjects’ first diabetes evaluation by the clinician, and the end point of follow-up was a new diabetes event. Follow-up visits were mainly made in physical examination centers, and the frequency was once a year.

The diagnosis of diabetes was defined as a self-reported history of diabetes or a measurement of FPG ≥ 7.00 mmol/L during follow-up [23]. For those who had been diagnosed with diabetes, the researchers again reviewed their blood glucose at the date of the diagnosis or at the last visit.

#### Drinking/smoking status

At the time of the baseline data access, according to their drinking/smoking history, participants were divided into four groups: non-drinking/smoking, former drinking/smoking, current drinking/smoking and not recorded.

BMI was calculated as weight/height^2^;

TyG was calculated as Ln [FPG (mg/dL) × TG (mg/dL)/2] [15];

TyG-BMI was calculated as TyG × BMI [18];

### Statistical analysis

R language software version 3.4.3 and Empower (R) software version 2.20 were used for data analysis. In this study, the baseline indicators were grouped according to the quintile of TyG-BMI, and the baseline characteristics of the proportion, median (interquartile range) or mean (SD: standard deviation) of individuals in each group were compared, in which TyG-BMI grouping was carried out by quantile function. The differences in categorical variables among the TyG-BMI groups were compared by Pearson’s χ2 test. The differences in continuous variables among the TyG-BMI groups were compared by ANOVA and Kruskal–Wallis H tests, and then Tukey’s HSD test and the Steel-Dwass test were used as post hoc tests.

Multivariate Cox regression was used to evaluate the relationship between diabetes and TyG-BMI, and the hazard ratio (HR) for diabetes and the corresponding 95 % confidence interval (CI) were calculated after controlling for confounding variables. Four models were used for multivariate Cox regression analysis in this study [24]: Model I (adjusted for the most basic demographic variables: sex, age and height), model II (adjusted for variables that contributed more than 10 % to the risk of TyG-BMI matching with diabetes), model III (adjusted model II + univariate analysis of variables with *P* < 0.05) and model IV (all non-collinear variables were adjusted) [25]. The Kaplan–Meier survival curve showed the risk of diabetes for each TyG-BMI quintile. At the same time, the area under the curve (AUC) was calculated by receiver operating characteristic (ROC) curve analysis, and the predictive abilities of the TyG index, BMI, FPG and TyG-BMI for diabetes risk were compared.

To further analyze the association between diabetes and TyG-BMI, the researchers also applied a hierarchical Cox regression model for hierarchical analysis to explore the association between TyG-BMI and diabetes in people of different ages, sexes and BMIs. Age stratification was performed according to a previous study [21], and BMI stratification was carried out according to the criteria recommended by the Chinese Obesity working Group [26]. The likelihood ratio test was used to examine the differences between different hierarchical groups to determine whether there were interactions.

## Results

### Anthropometric and biochemical characteristics of the study subjects

After excluding the subjects who did not meet the criteria, 116,661 eligible subjects were finally analyzed. Including 62,759 men and 53,902 women, their average age was 44.07 (12.93) years old, and their average TyG-BMI was 197.30 (36.96). The anthropometric and biochemical characteristics of patients who were stratified according to the TyG-BMI quintile are shown in Table 1. It is not difficult to see that in this study, the BMI, weight, age, systolic/diastolic blood pressure (S/DBP), height, FPG, TyG, total cholesterol (TC), aspartate aminotransferase (AST), triglyceride (TG), blood urea nitrogen (BUN), low-density lipid cholesterol (LDL-C), alanine aminotransferase (ALT) and serum creatinine (Scr) of the subjects gradually increased with the increase of TyG-BMI, while the HDL-C gradually decreased with the increase of TyG-BMI. Additionally, the proportion of men gradually increased with the gradual increase in TyG-BMI, while the proportion of women gradually decreased.

**Table 1:** Baseline characteristics of the study subjects

	TyG-BMI quintile					*P*-value
	Q1(96.68-164.00)	Q2(164.00-184.00)	Q3(184.00-204.10)	Q4(204.11-228.11)	Q5(228.11-477.08)	
No. of subjects	23332	23332	23333	23331	23333	
Age (years)	35.00 (31.00-42.00)	39.00 (33.00-49.00)	43.00 (35.00-54.00)	46.00 (36.00-57.00)	46.00 (37.00-58.00)	<0.001
Sex						<0.001
Male	5899 (25.28%)	9418 (40.37%)	13306 (57.03%)	16083 (68.93%)	18053 (77.37%)	
Female	17433 (74.72%)	13914 (59.63%)	10027 (42.97%)	7248 (31.07%)	5280 (22.63%)	
Family history of diabetes	492 (2.11%)	563 (2.41%)	522 (2.24%)	517 (2.22%)	540 (2.31%)	<0.001
Height (cm)	164.07 (7.44)	164.99 (8.13)	166.43 (8.49)	167.52 (8.39)	168.44 (8.30)	<0.001
Weight (kg)	52.02 (5.79)	58.69 (6.53)	64.41 (7.32)	70.03 (7.83)	79.24 (10.26)	<0.001
BMI (kg/m^2^)	19.29 (1.30)	21.50 (1.12)	23.19 (1.20)	24.89 (1.32)	27.86 (2.36)	<0.001
SBP (mmHg)	110.29 (13.49)	114.84 (14.86)	119.76 (15.65)	123.68 (16.16)	128.57 (16.60)	<0.001
DBP (mmHg)	68.00 (63.00-74.00)	70.00 (64.00-77.00)	74.00 (67.00-81.00)	76.00 (70.00-84.00)	80.00 (73.00-88.00)	<0.001
FPG (mmol/L)	4.69 (0.53)	4.83 (0.54)	4.94 (0.57)	5.05 (0.60)	5.23 (0.64)	<0.001
TG (mmol/L)	0.66 (0.51-0.84)	0.87 (0.69-1.11)	1.10 (0.85-1.45)	1.44 (1.10-1.90)	2.07 (1.51-2.88)	<0.001
TyG	7.81 (0.38)	8.11 (0.38)	8.38 (0.41)	8.67 (0.43)	9.08 (0.52)	<0.001
TC (mmol/L)	4.43 (0.79)	4.63 (0.83)	4.79 (0.87)	4.95 (0.89)	5.14 (0.92)	<0.001
HDL-C (mmol/L)	1.53 (0.31)	1.45 (0.29)	1.36 (0.28)	1.30 (0.27)	1.23 (0.27)	<0.001
LDL-C (mmol/L)	2.50 (0.59)	2.68 (0.63)	2.81 (0.67)	2.91 (0.68)	2.95 (0.72)	<0.001
ALT (IU/L)	13.00 (10.20-17.00)	15.00 (11.50-20.30)	18.00 (13.60-25.00)	22.00 (16.00-31.28)	29.00 (20.10-43.70)	<0.001
AST (IU/L)	19.90 (17.00-23.00)	20.70 (17.70-24.20)	22.00 (18.70-26.00)	23.00 (19.90-28.00)	25.70 (21.20-32.00)	<0.001
BUN (mmol/L)	4.28 (3.60-5.09)	4.44 (3.73-5.25)	4.60 (3.88-5.42)	4.72 (4.02-5.53)	4.79 (4.06-5.60)	<0.001
Scr (mmol/L)	60.30 (53.30-70.90)	64.70 (55.60-77.60)	70.90 (59.00-82.00)	74.90 (63.20-84.30)	76.10 (66.00-85.80)	<0.001
Smoking status						<0.001
Non	556 (2.38%)	859 (3.68%)	1199 (5.14%)	1719 (7.37%)	2325 (9.96%)	
Former	95 (0.41%)	172 (0.74%)	294 (1.26%)	362 (1.55%)	403 (1.73%)	
Current	5106 (21.88%)	5022 (21.52%)	4995 (21.41%)	4861 (20.83%)	4665 (19.99%)	
Not recorded	17575 (75.33%)	17279 (74.06%)	16845 (72.19%)	16389 (70.25%)	15940 (68.32%)	
Drinking status						<0.001
Non	50 (0.21%)	82 (0.35%)	151 (0.65%)	233 (1.00%)	356 (1.53%)	
Former	483 (2.07%)	759 (3.25%)	1160 (4.97%)	1426 (6.11%)	1696 (7.27%)	
Current	5224 (22.39%)	5212 (22.34%)	5177 (22.19%)	5283 (22.64%)	5341 (22.89%)	
Not recorded	17575 (75.33%)	17279 (74.06%)	16845 (72.19%)	16389 (70.25%)	15940 (68.32%)	

Values were expressed as mean (SD) or medians (quartile interval) or n (%). The differences among quintiles were evaluated by one-way ANOVA and Tukey’s HSD test or the Kruskal–Wallis test and Steel–Dwass test. After making a pairwise comparison between the quintiles, the results showed that there were significant differences among all groups (*P* < 0.05)

Abbreviations: *BMI* Body mass index, *SBP* systolic blood pressure, *DBP* diastolic blood pressure *FPG* fasting plasma glucose, *TG* triglyceride, *TyG* the triglyceride-glucose index, *TyG-BMI* triglyceride glucose-body mass index, *TC* total cholesterol, *LDL-C* low-density lipid cholesterol, *BUN* blood urea nitrogen, *Scr* serum creatinine, *ALT* alanine aminotransferase, *AST* aspartate aminotransferase

### Follow-up results of the subjects

**Fig. 2:**
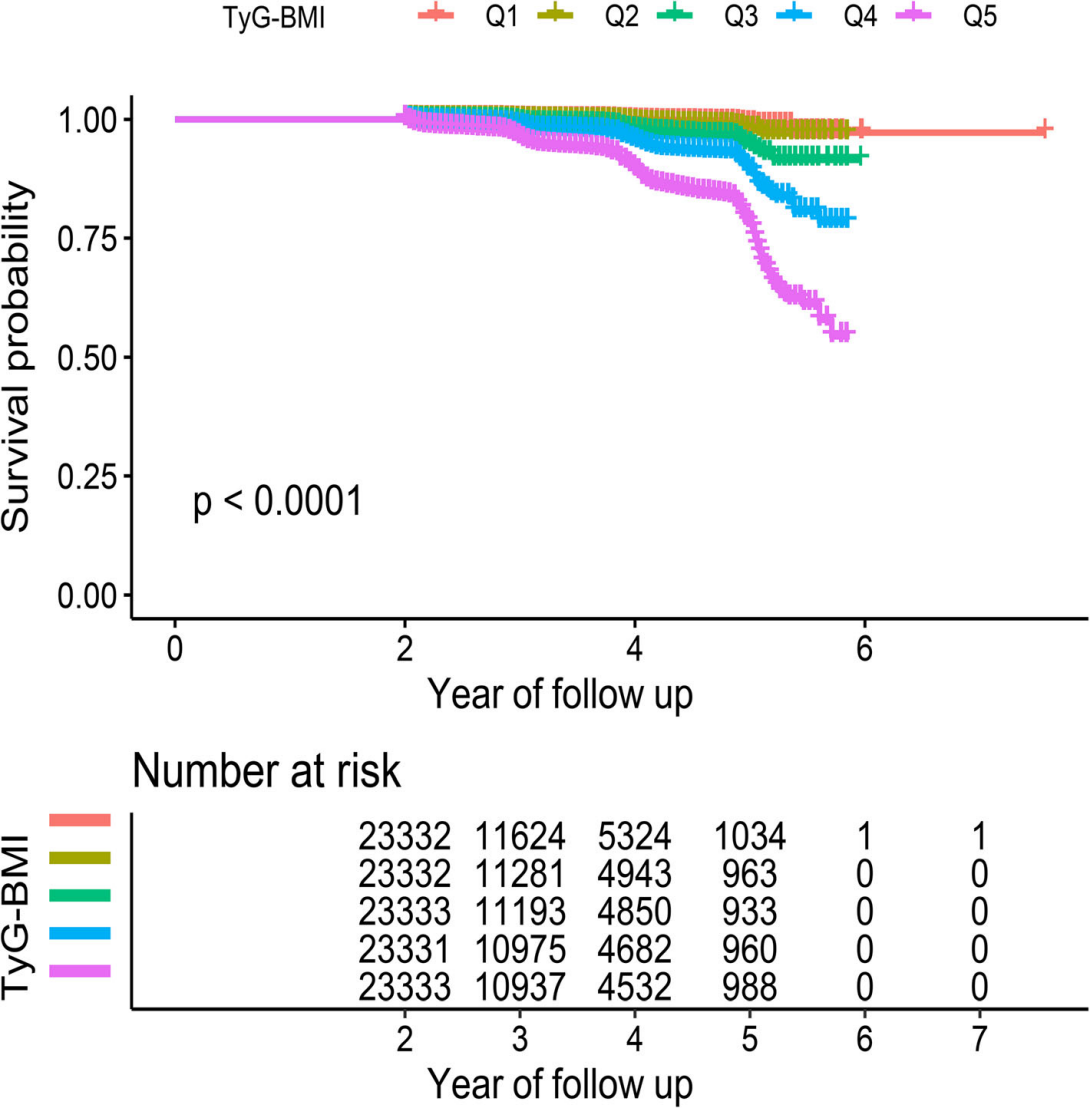
Kaplan-Meier analysis of future diabetes risk according to TyG-BMI quintiles. TyG-BMI: triglyceride glucose-body mass index

During the average follow-up period of 3.1 (0.95) years, 1888 men (1.61 %) and 793 women (0.68 %) were newly diagnosed with diabetes. The cumulative incidence of diabetes was 0.33 % (76/23,332) in Group Q1, 0.53 % (123/23,332) in Group Q2, 1.41 % (328/23,333) in Group Q3, 2.76 % (644/23,331) in Group Q4, and 6.47 % (1510/23,333) in Group Q5. Figure 2 shows the results of the Kaplan–Meier analysis based on the TyG-BMI quintile, in which the incidence of diabetes in the Q1 group was significantly higher than that in the other groups.

### Diabetes risk of TyG-BMI and its quintile

Cox regression models were established to evaluate the association between TyG-BMI and diabetes. Before modeling, the collinearity between the covariate and TyG-BMI was first checked [27]. Among them, body weight, BMI, TyG, TC and TyG-BMI had high collinearity and could not be included in the following analysis (Supplementary Table 1). In this study, four multivariate adjustment models were established (Table 2), in which Model I adjusted the covariates related to general demographics. The results indicated that TyG-BMI was strongly positively correlated with the risk of future diabetes (HR 2.12 per SD increase, 95 % CI: 2.05 to 2.19), and the risk of diabetes corresponding to TyG-BMI was gradually increased compared with the lowest quintile group (*P* for trend < 0.00001). Model II adjusted for variables that contributed more than 10 % to the risk of TyG-BMI matching with diabetes, and the results suggested that the association between TyG-BMI and diabetes was slightly weakened. After further adjusting for significant variables in the univariate analysis on the basis of model II (model III), the positive correlation between TyG-BMI and its quintile and diabetes remained stable (HR 1.50 per SD increase, 95 % CI: 1.40 to 1.60, *P* for trend < 0.00001). Finally, the researchers adjusted all non-collinear variables, and the results were similar to those before. In summary, TyG-BMI was an independent risk factor for new-onset diabetes.

**Table 2:** Hazard ratios for diabetes events by quintiles of TyG-BMI

	HR (95%CI)						
	Multivariable Analysis (per SD increase)	Q1	Q2	Q3	Q4	Q5	*P* for trend
Model I	2.12 (2.05, 2.19)	Ref	1.25 (0.94, 1.67)	2.82 (2.19, 3.63)	4.89 (3.84, 6.23)	11.11 (8.77, 14.06)	<0.001
Model II	1.46 (1.40, 1.52)	Ref	1.14 (0.85, 1.52)	2.33 (1.81, 3.00)	3.08 (2.41, 3.94)	4.62 (3.62, 5.89)	<0.001
Model III	1.50 (1.40, 1.60)	Ref	1.16 (0.74, 1.80)	2.05 (1.37, 3.07)	3.23 (2.19, 4.77)	4.69 (3.17, 6.94)	<0.001
Model IV	1.49 (1.40, 1.59)	Ref	1.15 (0.74, 1.80)	2.05 (1.37, 3.06)	3.22 (2.18, 4.75)	4.65 (3.14, 6.89)	<0.001

Model I adjusted for sex, age and height

Model II adjusted for SBP, FPG, HDL-C and ALT

Model III adjusted for age, sex, SBP, DBP, FPG, TG, HDL-C, LDL-C, ALT, AST, BUN, Scr, Smoking status, Drinking status and family history of diabetes

Model IV adjusted for age, sex, SBP, DBP, FPG, TG, HDL-C, LDL-C, ALT, AST, BUN, Scr, Smoking status, Drinking status family history of diabetes and height

Abbreviations: *TyG-BMI* triglyceride glucose-body mass index, *HR* hazard ratios, *CI* confidence, other abbreviations as in Table 1

### Association of TyG-BMI with new-onset diabetes in different subgroups

As stated in previous studies, TyG-BMI is a marker that can better reflect the metabolic state of the body [11, 12]. Therefore, the researchers continued to explore the association between TyG-BMI and diabetes in different sexes, ages and BMI phenotypes, and the results showed that there were significant differences in TyG-BMI-related diabetes risk among different ages and BMI (*P* for interaction < 0.05). As shown in Table 3, among people with different phenotypes, it can be observed that some special groups have a significantly higher risk of developing diabetes. Among them, in the age stratification, the risk of TyG-BMI-related diabetes in young and middle-aged people was significantly higher than that in middle-aged and elderly people [HR (per SD increase): 20-30years: 1.83, 31-40years: 2.16, 41-50years: 1.87 vs. 51-60years: 1.45, 61-70years: 1.14, > 70years:1.26]. In BMI stratification, non-obese people seem to be the focus of attention, and non-obese people had a significantly higher risk of TyG-BMI-related diabetes than overweight and obese people [HR (per SD increase): BMI < 24 kg/m^2^: 2.32 vs. BMI ≥ 24, < 28 kg/m^2^: 1.68, BMI ≥ 28 kg/m^2^: 1.25].

**Table 3:** Stratified association between TyG-BMI and diabetes by age, sex, and BMI

Subgroup	No. of participants	unadjusted HR (95 %CI)	adjusted HR (95 %CI)	*P* for interaction
Age (years)				0.0095
20–30	14,934	2.81 (2.34, 3.36)	1.83 (1.24, 2.70)	
31–40	41,461	2.79 (2.58, 3.01)	2.16 (1.90, 2.47)	
41–50	26,038	2.58 (2.41, 2.76)	1.87 (1.65, 2.12)	
51–60	19,151	2.06 (1.95, 2.19)	1.45 (1.31, 1.61)	
61–70	10,701	1.67 (1.55, 1.80)	1.14 (1.01, 1.29)	
>70	4376	1.56 (1.40, 1.74)	1.26 (1.08, 1.48)	
Sex				0.0651
Male	62,759	2.13 (2.05, 2.22)	1.44 (1.33, 1.55)	
Female	53,902	2.51 (2.39, 2.64)	1.59 (1.45, 1.74)	
BMI (kg/m^2^)				< 0.0001
<24	69,459	5.57 (4.92, 6.31)	2.32 (1.86, 2.89)	
≥24, < 28	37,086	3.47 (3.15, 3.82)	1.68 (1.38, 2.04)	
≥ 28	10,116	1.80 (1.65, 1.97)	1.25 (1.07, 1.45)	

Adjusted for age, sex, SBP, DBP, FPG, TG, HDL-C, LDL-C, ALT, AST, BUN, Scr, Smoking status, Drinking status and family history of diabetes

Abbreviations as in Tables 1 and 2

### Predictive value of TyG-BMI in new-onset diabetes

**Fig. 3:**
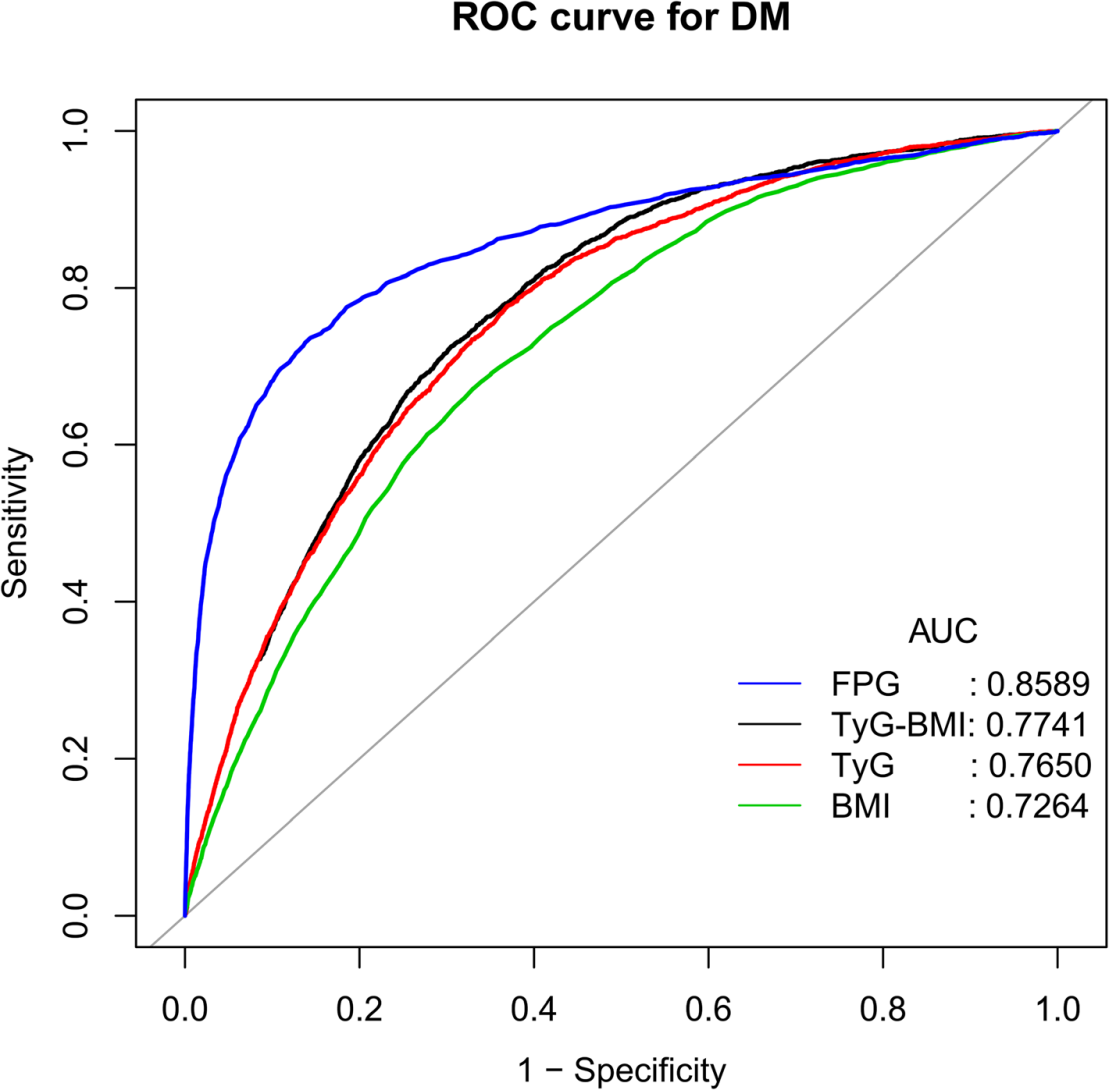
Receiver operating characteristic (ROC) curve analyses to predict diabetes. AUC: area under the curve; BMI: body mass index, TyG: triglyceride-glucose index, TyG-BMI: triglyceride glucose-body mass index

To compare the predictive value of TyG-BMI for new-onset diabetes, the researchers performed ROC curve analysis (Fig. 3). For the prediction of new-onset diabetes, the AUCs of TyG-BMI, BMI, FPG and TyG were 0.7741 (0.7658–0.7824), 0.7264 (0.7172–0.7355), 0.8589 (0.8504–0.8675) and 0.7650 (0.7564–0.7736), respectively. The AUC of TyG-BMI was significantly higher than that of BMI or TyG alone (both *P* < 0.001). The best cutoff value of TyG-BMI was 213.2966, the sensitivity was 72.51 %, and the specificity was 69.54 % (Table 4). However, in this study, TyG-BMI did not perform as well as FPG for predicting new-onset diabetes.

**Table 4:** Areas under the receiver operating characteristic curves for each evaluated parameters in identifying diabetes

Test	AUC	95 %CI low	95 %CI upp	Best threshold	Specificity	Sensitivity
TyG-BMI	0.7741	0.7658	0.7824	213.2966	0.6954	0.7251
TyG^*^	0.7650	0.7564	0.7736	8.5673	0.6312	0.7766
BMI^*^	0.7264	0.7172	0.7355	24.5950	0.6695	0.6718
FPG	0.8589	0.8504	0.8675	5.5050	0.8564	0.7363

*AUC* area under the curve; other abbreviations as in Table ​1

*P < 0.0001, compare with TyG-BMI

## Discussion

In this retrospective cohort study based on the Chinese population, it was observed that regardless of whether TyG-BMI was a categorical variable or a continuous variable, after fully adjusting for the covariates, TyG-BMI was always independently positively correlated with new-onset diabetes. ROC analysis suggested that TyG-BMI was superior to TyG and BMI in predicting new-onset diabetes.

### Comparisons with other studies and what does the current work add to the existing knowledge

Diabetes is a metabolic disease with serious health consequences, and early prevention and screening should be given full attention [1, 6]. As the testing method using HIEC technology is expensive and time-consuming, it has become a hot topic to identify simple and practical clinical markers. In this context, a large number of obesity-related parameters and anthropometric parameters have been studied. TyG-BMI is the product of TyG and BMI, which was first reported by Professor Er in 2016 [18]. After comparing traditional lipid parameters, blood glucose parameters, blood lipid ratio and obesity-related indicators, they found that TyG-BMI had the highest AUC for identifying IR. In addition, in several recent studies, TyG-BMI has been found to be closely related to prehypertension, NAFLD and stroke [28–30]. These results suggest that TyG-BMI has value as a predictor of metabolic diseases.

In this study, the association strength between TyG-BMI and diabetes risk was smaller than that of two similar previous studies [19, 20], which may be related to the more fully adjusted model in this study. In a cross-sectional study conducted in China in 2016, Zheng et al. evaluated the relationship between diabetes and TyG-BMI for the first time [19]. Their results indicated that after adjusting for DBP, age, SBP and sex, the OR for diabetes in the quartile of TyG was 1, 2.41, 3.79 and 9.04, respectively. Another follow-up study from Spain confirmed this finding [20]. They adjusted covariates such as age, smoking, drinking and physical activity in the logical regression model. The OR value of diabetes corresponding to TyG-BMI was 4.63 (3.12–6.89) in the fourth quartile. Different from these two cross-sectional studies, this study adopted a longitudinal design and further expanded the sample size, confirming that there is a causal association between TyG-BMI and diabetes, which is independent of traditional risk factors.

Some interesting phenomena were also found in the subgroup analysis of this study, in which there were significant differences in TyG-BMI-related NAFLD risk among different ages and BMI phenotypes. In the subgroup of age stratification, the risk of TyG-BMI-related diabetes in young and middle-aged people was significantly higher than that in middle-aged and elderly people. This strange phenomenon is considered to be the impact of the rapid development of society. At present, in China and around the world, the aging population is increasing, and the long-term birth control policy has led to a shrinking labor force [31–33], further aggravating the social pressure on young and middle-aged people [34, 35]. In the BMI stratified subgroup, non-obese people actually have a higher risk of TyG-BMI-related diabetes than overweight and obese people, which seems abnormal, but diabetes in non-overweight individuals is a problem that is receiving increasing attention in society [36]. With changes in social structure, lifestyle and diet, some subtle changes have taken place in the human body structure, especially fat storage, which has increased significantly [37, 38]. Relying solely on BMI to distinguish obesity cannot reflect this information [39].

Although the underlying mechanism of the relationship between TyG-BMI and diabetes is unclear, it may be related to IR. IR is the core mechanism of the occurrence and progression of diabetes, which has been confirmed in previous studies [1]. TyG-BMI is a combined marker of FPG, TG and BMI, and the role of TG and FPG in the identification of IR has been well verified in previous studies [40, 41]. Among people with normal blood glucose levels, a higher level of FPG is an independent risk factor for diabetes. When FPG increases gradually, the insulin sensitivity of skeletal muscle decreases [40, 42]. On the other hand, hepatic TG content is an important determinant of hepatic IR [43]. A combination of TG and FPG has a high sensitivity, similar to the HIEC test. After further combining it with BMI [16], its ability to identify IR is further improved [18].

This is the first study to explore the causal relationship between diabetes and TyG-BMI, and the results of this study provide a reliable marker for the early identification of individuals at high risk of diabetes. Although the attraction of using HIEC technology to measure IR is undeniable, it is not applicable to general physical examinations and large-scale epidemiological investigations considering its high economic and time cost [14], and the use of simple and effective alternative markers can better achieve the goals [18]. TyG-BMI is the product of TyG and BMI, which is fast and convenient to calculate and can better reflect the IR status [18]. In addition, indicators such as FPG, TG and BMI are very common and routine examination items, which provides greater convenience for the smooth development of population physical examinations and epidemiological studies.

### Study strength and limitations

The biggest strength of this study is that it includes a large sample of more than 100,000 people from many regions of China; after sufficient model adjustment, the independent relationship between diabetes and TyG-BMI has been confirmed. ROC analysis further confirmed that TyG-BMI was a better independent predictor of diabetes, and subgroup analysis identified high-risk populations. Through these reliable statistical analyses, the conclusion of this study can be considered to be quite reliable, and its findings can be applied to the majority of the Chinese population for the early assessment of diabetes risk.

The advantages of this study are clear, but limitations also exist, which are mainly as follows: (1) The diagnosis of diabetes in this study does not distinguish between type 2 diabetes and type 1 diabetes. However, the findings of this study may be more suitable for predicting the risk of type 2 diabetes because the number of patients with type 2 diabetes exceeds 95 % of all cases of diabetes [44]. (2) The diagnosis of diabetes in this study depends on the subjects’ self-report or FPG > 7.0 mmol/L during the follow-up period, which may lead to an underestimation of the true prevalence of diabetes. (3) Because this study is a post hoc analysis of a previous study [21], the database variables are fixed. Although many confounding factors were adjusted, there were still some variables that were not included in the database, such as IR, glycosylated hemoglobin, exercise habits, etc. Therefore, there may be some residual confounding [45]. (4) The patients were followed up for a relatively short period of time, and the immediate effect of a shorter follow-up time is a lower incidence of observed endpoint events. However, the researchers still found a strong correlation between the two, which suggests that TyG-BMI may have high value as a predictor of diabetes.

## Conclusions

This cohort study of the Chinese population shows that after excluding other confounding factors, there is a causal association of TyG-BMI with diabetes, and this independent association is more obvious in young, middle-aged and non-obese people. These data provide strong evidence to enhance the value of TyG-BMI in the evaluation of diabetes and to provide a simple and economical approach for the early prevention and treatment of diabetes.

## Supplementary Information

**Additional file 1: Supplementary Table 1.** Collinearity diagnostics steps.

## Data Availability

The data used in this study have been uploaded to the “Dryad” database by Professor Chen et al.

## Abbreviations

IR: Insulin resistance
TyG-BMI: Triglyceride glucose-body mass index
HIEC: Hyperinsulinemic-euglycemic clamp
TyG: Triglyceride glucose
TG: Triglyceride
FPG: Fasting plasma glucose
BMI: Body mass index
HR: Hazard ratio
CI: Confidence interval
AUC: Area under the curve
ROC: Receiver operating characteristic curve
S/DBP: Systolic/diastolic blood pressure
TC: Total cholesterol
TG: Triglyceride
LDL-C: Low-density lipid cholesterol
ALT: Alanine aminotransferase
AST: Aspartate aminotransferase
BUN: Blood urea nitrogen
Scr: Serum creatinine
SD: Standard deviation
