# Supplementary material for: Triglyceride glucose-body mass index and the risk of diabetes: a general population-based cohort study
Source: Lipids Health Dis. 2021 Sep 6;20:99. doi: 10.1186/s12944-021-01532-7 (PMC8420033; doi:10.1186/s12944-021-01532-7)
Supplement: Supplementary file 1 — Additional file 1: Supplementary Table 1. Collinearity diagnostics steps. [file 12944_2021_1532_MOESM1_ESM.docx]

Supplementary Table 1: Collinearity diagnostics steps.

|  | VIF | | | | |
| --- | --- | --- | --- | --- | --- |
|  | Step 1 | Step 2 | Step 3 | Step 4 | Step 5 |
| TyG-BMI | 417.7 | 135.1 | 2.6 | 2.3 | 2.3 |
| Age | 1.5 | 1.5 | 1.5 | 1.4 | 1.4 |
| Sex | 3.2 | 3.2 | 3.2 | 3.1 | 3.1 |
| Height | 51.8 | 41.8 | 2.1 | 2.1 | 2.1 |
| Weight | 164.7 | 132.3 | NA | NA | NA |
| BMI | 294.7 | NA | NA | NA | NA |
| SBP | 2.2 | 2.2 | 2.2 | 2.2 | 2.2 |
| DBP | 2 | 2 | 2 | 2 | 2 |
| FPG | 1.4 | 1.4 | 1.4 | 1.2 | 1.2 |
| TyG | 59.4 | 24.1 | 7 | NA | NA |
| TC | 7 | 7 | 7 | 7 | NA |
| TG | 5.8 | 5.3 | 5.1 | 2 | 1.7 |
| HDL-C | 1.5 | 1.5 | 1.5 | 1.5 | 1.2 |
| LDL-C | 5.8 | 5.8 | 5.8 | 5.8 | 1.1 |
| ALT | 3.3 | 3.3 | 3.3 | 3.3 | 3.3 |
| AST | 3 | 3 | 3 | 3 | 3 |
| BUN | 1.2 | 1.2 | 1.2 | 1.2 | 1.2 |
| Cr | 2 | 2 | 2 | 2 | 2 |
| Smoking status | 3.1 | 3.1 | 3.1 | 3.1 | 3.1 |
| Drinking status | 3.1 | 3.1 | 3.1 | 3.1 | 3.1 |
| Family history of diabetes | 1 | 1 | 1 | 1 | 1 |

variance inflation factor; VIF = 1/(1-R^2^). Abbreviations as in Table ​1.

Note: The variables with VIF>5 will be regarded as collinear variables and cannot be included in the multiple regression model
